# Development and Validation of an Automated Zone Fluidics-Based Sensor for In Vitro Dissolution Studies of Captopril Using Total Error Concept

**DOI:** 10.3390/molecules26040824

**Published:** 2021-02-05

**Authors:** Theano D. Karakosta, Paraskevas D. Tzanavaras, Constantinos K. Zacharis

**Affiliations:** 1 LifeLabs Medical Laboratories, Toronto, ON M9W6J6, Canada; karakostatheano@hotmail.com; 2 Laboratory of Analytical Chemistry, Department of Chemistry, Aristotle University of Thessaloniki, 54124 Thessaloniki, Greece; 3 Laboratory of Pharmaceutical Analysis, Department of Pharmaceutical Technology, School of Pharmacy, Aristotle University of Thessaloniki, 54124 Thessaloniki, Greece

**Keywords:** captopril, zone fluidics, dissolution, accuracy profiles, validation

## Abstract

In the present research, a zone fluidics-based automated sensor for the analysis of captopril in in vitro dissolution samples is reported. Captopril is reacted under flow conditions with Ni(II) (10 mmol L^−1^) in alkaline medium (0.15% *v/v* NH_3_) to form a stable derivate, which is monitored spectrophotometrically at 340 nm. The chemical and instrumental parameters were carefully investigated and optimized. The validation of the developed method was performed in the range of 5 to 120% of the expected maximum concentration using the accuracy profiles as a graphical decision-making tool. The *β*-expectation tolerance intervals did not exceed the acceptance criteria of ±10%, which means that 95% of future results will be encompassed in the defined bias limits. The variation of the relative bias ranged between −2.3% and 3.5% and the RSD values for repeatability and intermediate precision were lower than 2.3% in all cases. The limit of detection (LOD), and the lower and the upper limit of quantification (LLOQ, ULOQ) were satisfactory and found to be 1%, 5% and 120% (corresponding to 0.6, 2.78 and 66.67 μg mL^−1^ in dissolution medium). The developed method was successfully applied for the analysis of captopril in dissolution tests of two commercially available batches.

## 1. Introduction

Captopril (CAP), (2S)-1-[(2S)-2-methyl-3-sulfanylpropanoyl)] pyrrolidine-2-carboxylic acid, is an antihypertensive drug that is administered orally. CAP is included in Class III of the Biopharmaceutical Classification System (BCS) characterized by high solubility and low membrane permeability. It therefore offers decreased absorption rate, whereas dissolution occurs very rapidly. Following a single oral dose, the elimination half-life ranges from approximately 1.7 to 2 h. From a chemical point of view, CAP, as a thiol, is stable in an acidic medium. At elevated pH values, it becomes unstable and undergoes oxidative degradation of the R-SH group. In vivo, CAP is extensively converted to metabolites such as mixed disulfide conjugates with either low molecular weight endogenous thiols (cysteine and glutathione) or with proteins [1,2,3].

Dissolution testing is a critical tool both during new drug development and for quality control testing of commercial formulations during production [4,5,6]. From an analytical chemistry point of view, dissolution testing produces a large amount of samples, particularly when time based dissolution profiles have to be created. Rapid analysis of dissolution samples is particularly important under an industrial QC environment during the production of commercially available formulations. Analysis of dissolution samples is typically carried out using HPLC through methods proposed by international pharmacopoeias. Alternatively, in several cases, direct UV-vis analysis is suggested. Although the latter approach is the simplest and fastest in terms of throughput, exhaustive validation should prove its selectivity against the pharmaceutical excipients and the dissolution medium, particularly when measurements are carried out at the low UV region (<250 nm). Another issue that has to be taken under consideration is that re-validation in terms of selectivity is necessary when different brands (containing different excipients) are processed. On the other hand, HPLC offers enhanced selectivity through its separation principles and a more generic brand-to-brand character; however, it is significantly more time consuming and does not allow real time flow through monitoring.

On the basis of automation of chemical analysis, the concept of zone fluidics (ZF) offers several advantages that are well-established in the literature and are useful in the QC of pharmaceuticals [7,8,9]. A detailed review discussing automated flow methods for the determination of CAP was published in 2011 [10]. More recent published automated flow methods include the use of nitroprusside as spectrophotometric reagent [11], immobilized AgSCN on polyurethane resin (indirect method [12]), Ce(IV)-based chemiluminescence [13] and automated solid phase extraction coupled to HPLC with post column derivatization for urine analysis [14].

In the present study, we report the automation of a new color-forming reaction for the rapid and reliable determination of CAP in samples produced from in vitro dissolution studies. The developed method is based on the 2:1 complex formation between CAP and Ni(II) in alkaline medium [15,16]. The reaction is simple, rapid and utilizes readily available reagents. The selectivity of the proposed “chemistry” is superior compared to the USP-recommended UV spectrophotometric method (at 205 nm). The high sampling throughput (almost 25 samples h^−1^) of the reported ZF-approach is an important advantage compared to the HPLC one. Additionally, compared to previously reported automated methods, it does not require complicated SPE procedures [12,14] and it utilizes readily available UV-Vis detection [13]. The proposed analytical protocol was thoroughly validated according to the “*Société Française des sciences et techniques pharmaceutiques*” (SFSTP) harmonization guidelines. This protocol is based on the usage of the accuracy profiles, which take into account the total error (systematic and random errors of the results). The method was used to support the dissolution studies of CAP from two commercial formulations.

## 2. Results and Discussion

### 2.1. Preliminary Experiments

Preliminary experiments using a three-zones approach confirmed that the Ni(II)–CAP complex could be formed under zone fluidics conditions. However, the order of mixing of the zones proved to be critical. Aspiration and direct contact of the Ni(II) and NH_3_ solution zones resulted in a dramatic decrease of the sensitivity of the reaction. Apparently the formation of a complex between Ni(II) and NH_3_ inhibits the colour-forming reaction. A series of tests revealed that the optimum order of mixing of the zones is Ni(II)/CAP/NH_3_. In this case, the direct contact of Ni(II) and ammonia is avoided and the formation of the Ni(II)–CAP complex is favoured. These findings are in accordance with previous reports on the spectral characteristics of the complex [15,16].

Preliminary experiments also confirmed the sensitizing effect of NH_3_ on the formation of the complex. Sodium hydroxide solutions at the same pH resulted in almost 2-fold lower signals.

### 2.2. Optimization of ZF Variables

Chemical and instrumental variables were investigated by the one-factor-at-a-time (OFAT) approach. The examined variables included the concentrations of Ni(II) (C(Ni(II)) and ammonia (C(NH_3_)), the flow rate (Q_v_) towards the detector, the volume of the sample (V_S_) and the length of the reaction coil. All experiments were carried out at ambient temperature using a volume of 50 μL of both Ni(II) and NH_3_ solutions.

**Figure 1:**
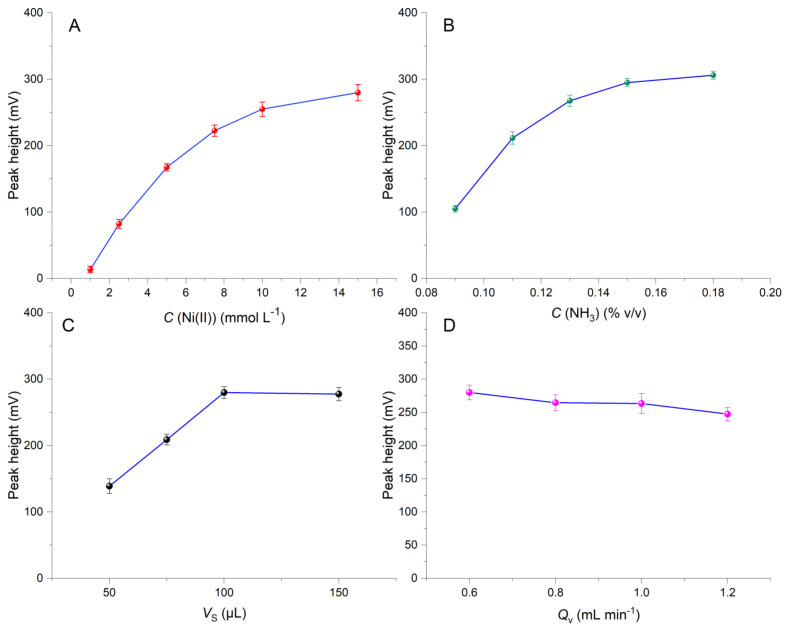
Effect of the (**A**) Ni(II) concentration (*C*(Ni(II)), (**B**) NH_3_ concentration (*C*(NH_3_)), (**C**) sample volume (*V*_s_) and (**D**) flow rate (*Q*_v_) on the sensitivity of the proposed ZF method.

The effect of the concentration of Ni(II) was examined in the range of 1 to 15 mmol L^−1^. As can be seen in the experimental results of Figure 1A, a non-linear 20-fold increase in the signals was observed in the range of 1–10 mmol L^−1^ with the phenomenon being less pronounced thereafter. Based on these findings, the value of 10 mmol L^−1^ was selected for further studies. A similar, non-linear, behavior was also observed by increasing the volume fraction of NH_3_ in the range of 0.09 to 0.17% *v/v* (Figure 1B). The signals practically leveled-off for values higher than 0.15% *v/v*, which was selected for the subsequent experiments.

The effect of the sample injection volume is, in most cases, critical in terms of sensitivity since it directly affects the dispersion of the sample in the ZF configuration. As can be seen in the results of Figure 1C, the signals increased linearly in the range of 50–100 μL CAP and leveled-off in the range of 100–150 μL. Finally, an injection volume of 100 μL was selected as optimal.

The investigation of the effects of the flow rate towards the detector and of the length of the reaction coil confirmed the rapidity of the reaction and therefore its suitability for on-line applications. As illustrated in Figure 1D, an increase of the coil length in the range of 30 to 90 cm resulted in a linear decrease in the sensitivity due to the dispersion effect on the mixed zones. Based on these results, the shorter length from a practical point of view (at 30 cm) was therefore selected for subsequent experiments. In ZF single-channeled configurations, the flow rate towards the detector determines the reaction time and the mixing efficiency of the zones. In our case, the flow rate showed negligible impact on the sensitivity in the range of 0.6–1.2 mL min^−1^, confirming fast reaction kinetics and adequate mixing of the zones. A reasonable value of 0.9 mL min^−1^ was finally selected.

### 2.3. Method Validation

The primary role of validating analytical methods is to prove that the method is suitable for its intended use, fulfilling the anticipations described in ICH Q2(R1) guidelines [17]. The developed ZF-based analytical method was validated by implementing the accuracy profiles as a decision tool according to the SFSTP Commission proposal.

#### 2.3.1. Selectivity

The selectivity of the proposed method assay was evaluated in order to investigate the potential effects of the declared excipients (colloidal silicon dioxide, pre-gelatinized starch, magnesium stearate, titanium dioxide, sodium saccharin, sodium citrate, microcrystalline cellulose, polyvinyl pyrolidone, hydroxypropyl cellulose and gelatin) on the determination of captopril at the expected concentration of 55.5 μg mL^−1^ (100% of the theoretical concentration). The placebo mixture was prepared by mixing 1 g of each excipient in a mortar until a homogenous fine powder was obtained. An appropriate amount of the placebo mixture (at a ratio of 200 mg placebo per 50 mg CAP) was dispersed in captopril standard and sonicated for 10 min. The obtained solution was centrifuged at 4000 rpm for 10 min and then filtered through a 0.45 μm syringe filter prior to analysis. No interferences were observed from the excipients either individually or as a placebo mixture.

Additionally, 200 mg of placebo were subjected to a dissolution experiment for 30 min, and filtered samples were analyzed. We estimated the potential interference from the placebo according to the USP recommendations/formula to be <1.5% [18].

#### 2.3.2. Selection of the Response Function

The proposed method was validated using the total measurement error (systematic and random error) by constructing the accuracy profiles as a graphical decision tool in order to prove the suitability of the proposed method for its intended use [19]. This concept additionally involves the selection of the most appropriate calibration model for the determination of CAP and the range over which the method can be considered as valid. In order to find out the suitable response model, the fitting of different regression models to the calibration standards were investigated. The mean relative bias, the intermediate precision (*s*_r_ %), the upper and the lower *β*-ETI were calculated by using the back-calculated concentrations of the validation standards through each regression model. The suitability of these models was examined by plotting the accuracy profiles at a probability of 95%. The acceptance limits of *λ* at the ±10% level were considered.

**Figure 2:**
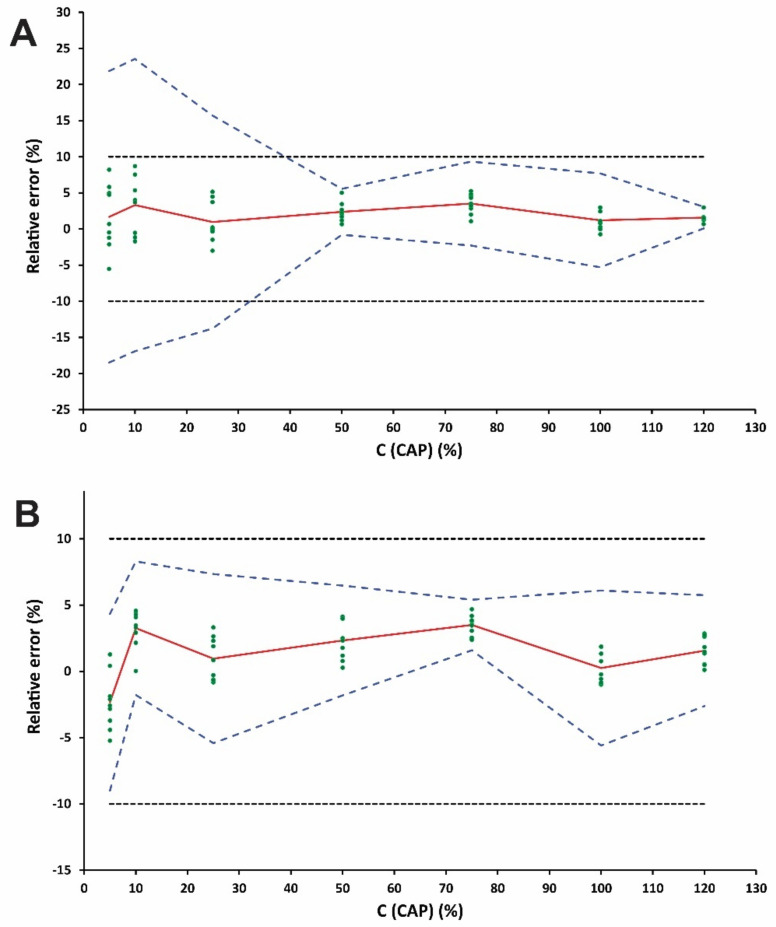
Accuracy profiles for the CAP determination using a (**A**) linear unweighted regression model using one standard at the highest concentration level (120%) and (**B**) linear unweighted regression model. The red plain, blue dashed and blank dotted lines correspond to the relative error (%), the accuracy profile and the acceptance limits *λ* (±10%), respectively.

**Figure 3:**
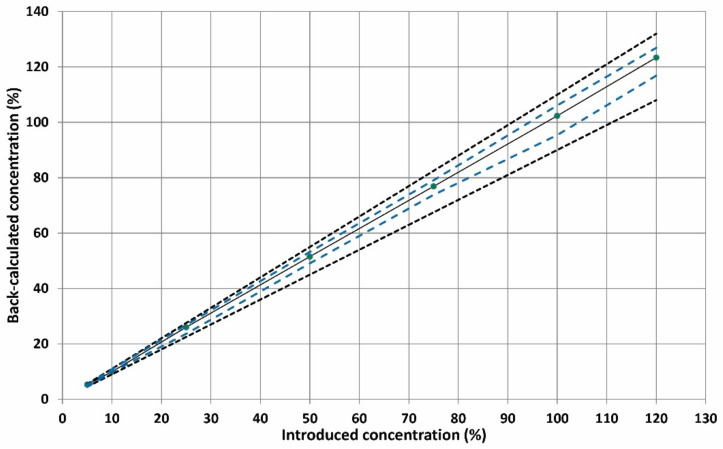
Linearity profile for CAP determination. The plain blank line corresponds to the identity line (Y = X), the blue dashed line represents the accuracy profile (*β*-ΕΤΙ) and the dotted curves illustrate the acceptance limits λ ± 10% expressed in the % concentration.

The linearity was assessed by fitting the experimental data (back-calculated concentrations and introduced concentrations) with the two simpler linear unweighted regression model in the specified calibration range. In the case of linear regression through the origin and one standard at the maximum concentration level of 120%, the scattering of the results led to a widening of the *β*-interval that could overpass or be higher than the acceptance limit, especially at lower concentration levels (Figure 2A). By applying linear regression (Figure 2B), the tolerance interval was entirely inside the acceptance limits for all examined concentration levels, offering sufficient performance in terms of trueness and precision even at the lowest calibration level of 5%. The validation results are tabulated in Table 1. The linearity of the method is depicted in Figure 3. The slope and intercept were found to be close to 1 and zero respectively while the R^2^ value was 0.9999, indicating good linearity of the developed ZF method.

#### 2.3.3. Trueness, Precision and Accuracy

Trueness is defined as the closeness of a mean value obtained from a set of replicated measurements, a conventionally accepted value [20]. This parameter was expressed as relative bias (%) and derived from the concentration of the validation standards after back-calculation. As reported in Table 1, the relative biases for each concentration level for CAP determination ranged between −2.3 and 3.5%, indicating a high trueness of the method.

The dispersion among replicated analyses is defined as precision and is described by the repeatability (intra-day) and the intermediate precision of a method (sum of intra-day and inter-day variances). Precision was expressed as the RSD of repeatability (*s*_r_, %) and time-dependent intermediate precision (*s*_R_, %) for each concentration level. According to Table 1, the *s*_r_ and *s*_R_ values were lower than 1.7 and 2.3% respectively, demonstrating adequate precision of the analytical scheme.

The accuracy is the closeness of agreement among the obtained results and a conventional true or accepted reference value as per the ICH Q2(R1) guideline [17]. The accuracy is based on the sum of systematic and random errors on the test result. As Figure 1B shows, the upper and lower *β*-ETIs for each concentration level for CAP (5 to 120%, corresponding to 2.78 to 66.67 μg mL^−1^) are entirely included inside the acceptance limit of ±10%. Therefore, the proposed analytical scheme can be considered as accurate in the studied range.

#### 2.3.4. Linearity, LODs and LOQs

The linearity of the method was investigated by fitting a least squares regression line on the back-calculated concentrations of the validation standards as a function of the introduced captopril concentration. Calibration parameters (slope, intercept, correlation coefficient, etc.) are tabulated in Table 1. The linearity of the proposed method was also proven because the absolute *β*-expectation tolerance limits were within the absolute acceptance limits (see Figure 3). The limit of detection (LOD) is considered to be the smallest analyte quantity that can be detected but not accurately quantified. The LOD was found to be 1% (corresponding to 0.6 μg mL^−1^ CAP) based on the following equation:

LOD = 3.3 × SD_b_/s

where SD_b_ is the standard deviation of the intercept and s is the slope of the regression line.

The lower and the upper limit of quantification (LLOQ, ULOQ) were obtained from the accuracy profiles and corresponding to the minimum and maximum concentration at which *β*-expectations intervals fall outside the acceptance limits. In our case, the *β*-expectation intervals are included inside the acceptance limit and therefore the LLOQ and ULOQ were 5% and 120% of the maximum expected concentration upon the complete dissolution.

#### 2.3.5. Robustness

The ability of the method to remain unaffected by small deliberate variations of the critical chemical and instrumental parameters is of key importance when developing and validating a new analytical approach for the quality control of pharmaceuticals. The selected parameters were: the flow rate towards the detector (0.8–1.0 mL min^−1^), the concentrations of the Ni(II) reagent (C(Ni(II)) = 9.5–10.5 mmol L^−1^) and NH_3_ solution (C(NH_3_) = 0.145–0.155% *v/v*), the injection volume of the sample (V_S_ = 95–105 μL) and the UV wavelength (λ = 338–342 nm). The experimental data indicated that in all cases the variation of the aforementioned variables caused a relative error of less than ±5% compared to the optimal conditions verifying the satisfactory robustness of the proposed automated analytical scheme.

### 2.4. Application in Dissolution Studies

**Figure 4:**
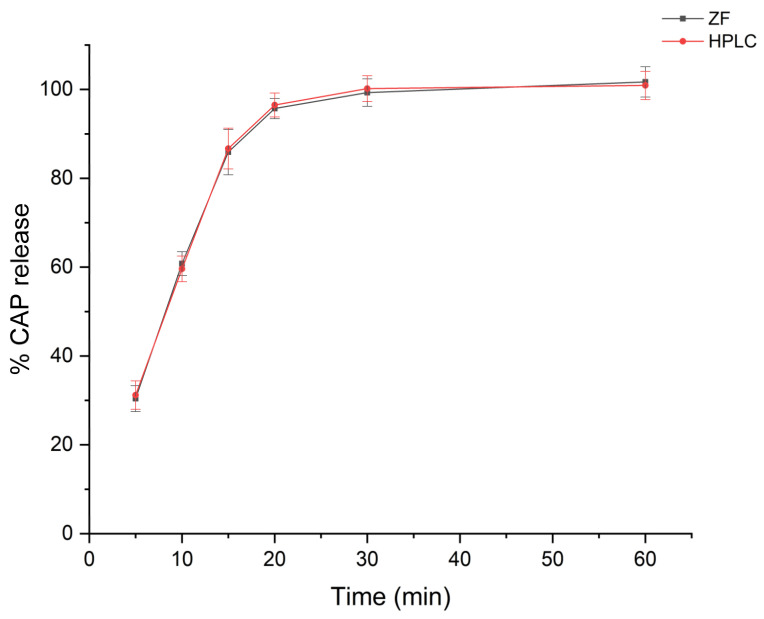
Comparison of the dissolution profile of captopril (Brand A) in 0.01 M HCl (Τ = 37.0 ± 0.5 °C, V = 900 mL, rotation speed = 50 rpm, n = 12) using the proposed ZF and HPLC methods.

The dissolution tests of CAP-containing tablets (50 mg/tab) were carried out in a type I dissolution apparatus as described in the US Pharmacopoeia [21]. Experimental conditions included 900 mL of 0.01 mol L^−1^ HCl as medium at 50 rpm paddles rotation speed. The temperature was kept constant at 37.0 ± 0.5 °C. Not less than 12 units (tablets) were processed for each batch or brand. Dissolution profiles were prepared by sampling at 5, 10, 15, 20, 30 and 60 min. According to the USP specifications, not less than 80% of the labeled amount of the active pharmaceutical ingredient (API) should be liberated within 20 min. On-line filtration was performed during collection through 45 μm PTFE discs and the samples were analyzed directly without additional processing. The dissolution medium was degassed ultrasonically for 15 min prior to usage. The dissolution profiles of two commercially available CAP formulations are depicted in Figure 4. Rapid dissolution of the formulations was confirmed based on the class III classification of the API according to BCS. In both cases the *Q* + 5% criterion was met.

Both formulations were also processed by the HPLC method proposed in the USP monograph for CAP [21]. The experimental results for both brands (ZF and HPLC) are included in Table 2. All results were within QC-accepted limits (*Q* > 80 and > *Q* + 5% after 20 min) and in good agreement with the USP HPLC method as *p*-values were > 0.05 (paired *t*-test, 95% confidence interval).

## 3. Materials and Methods

### 3.1. Reagents, Solutions and Materials

All reagents used in the present study were of the highest analytical grade and were purchased from Sigma-Aldrich (Life Science Chemilab S.A., Athens, Greece). Ultrapure water (18 MΩ cm resistivity) was used throughout this study (Millipore Direct-Q UV, Millipore S.A.S., Molsheim, France).

Nickel (II) reagent solution was prepared at the concentration level of 10 mmol L^−1^ by appropriate dissolution of NiCl_2_ hexahydrate in water. Aqueous ammonia solutions were also prepared daily at the required volume fractions by serial dilutions of the concentrated ammonia reagent (25% NH_3_ solution).

The placebo mixture used for method validation was prepared at a nominal concentration of 10 mg mL^−1^ from pharmaceutical grade excipients (colloidal silicon dioxide, pre-gelatinized starch, magnesium stearate, titanium dioxide, sodium saccharin, sodium citrate, microcrystalline cellulose, polyvinyl pyrolidone, hydroxypropyl cellulose and gelatin). Samples were filtrated through disposable PTFE syringe filters (0.45 μm) (Whatman^®^, GE Healthcare, Athens, Greece) prior to their analysis.

Captopril (>98%) stock standard solutions at a concentration of 1000 μg mL^−1^ were prepared in the dissolution medium (0.01 mol L^−1^ HCl). Working standard solutions were prepared daily by appropriate dilutions of stock standard in the same solvents. For method development, a captopril standard solution of 25 μg mL^−1^ was prepared.

### 3.2. Solutions for Method Validation

Calibration CAP standards at seven concentration levels (m = 7) ranging from 2.78 to 66.67 μg mL^−1^ (corresponding to 5–120% of the maximum expected captopril concentration upon the complete dissolution of a tablet) were performed in triplicate (n = 3) for each series of experiments (k = 3). The number of levels was adequate to produce different regression models. The validation standards comprised placebo mixture in dissolution medium (200 mg placebo per 900 mL of 0.01 mol L^−1^ HCl) spiked with known concentrations of captopril and were obtained from independent stock solutions. Three series of standards were made by spiking the exact volume of the stock solution of the analyte in order to obtain final concentrations of 2.78, 5.55, 13.89, 27.78, 41.66, 55.56 and 66.67 μg mL^−1^ (corresponding to 5, 10, 25, 50, 75, 100 and 120%) (m = 7) of the analyte, respectively. Three replicates (n = 3) were made per concentration level. To avoid potential carry over effects all glassware used in this study was previously washed with water.

### 3.3. Instrumentation and Apparatus

The ZF setup consisted of the following parts: a peristaltic pump (Minipuls3, Gilson), a micro-electrically actuated 10-port valve (Valco) and a flow-through UV-Vis spectrophotometric detector (λ_max_ = 340 nm, SPD-10A VP, Shimadzu). All necessary flow connections were made of 0.5 i.d. PTFE tubing, except for the holding coil (HC) that was made of 0.7 mm i.d. PTFE tubing. The reaction coil (RC) had the minimum length of 30 cm (0.5 mm i.d.). Operation and control was accomplished through a program that has been developed in house (LabVIEW, National Instruments^®^). Peak heights were used for data acquisition in all cases (Clarity^®^ software, version 4.0.3, DataApex).

In vitro dissolution experiments were carried out at the facilities of CosmoPharm Ltd (Greece) using a Distek Premier 5100 apparatus equipped with an autosampler (Type I). At each predetermined time, the sample was withdrawn from the dissolution vessel, filtered on-line and transferred to 5 mL vials for subsequent analysis by the proposed ZF method. In order to find out the optimum wavelength, off-line UV-Vis spectra were recorded using a UV-1601 batch spectrophotometer (Shimadzu, Kyoto, Japan).

The USP HPLC corroborative method was executed using an HP 1100 HPLC instrument from Agilent Technologies (Palo Alto, CA, USA). It comprised a quaternary pump (G1311A), a vacuum degasser (G1322A), a column thermostat (G1316A), an autosampler (G1313A) and a DAD spectrophotometric detector (G1315A). Chromatographic parameters (peak areas, retention times, theoretical plates etc) were calculated via the ChemStation^®^ software. A Hypersil BDS C_18_ (250 × 4.6 mm i.d.) column (packing L1) was employed.

### 3.4. ZF Procedure for the Determination of CAP

**Figure 5:**
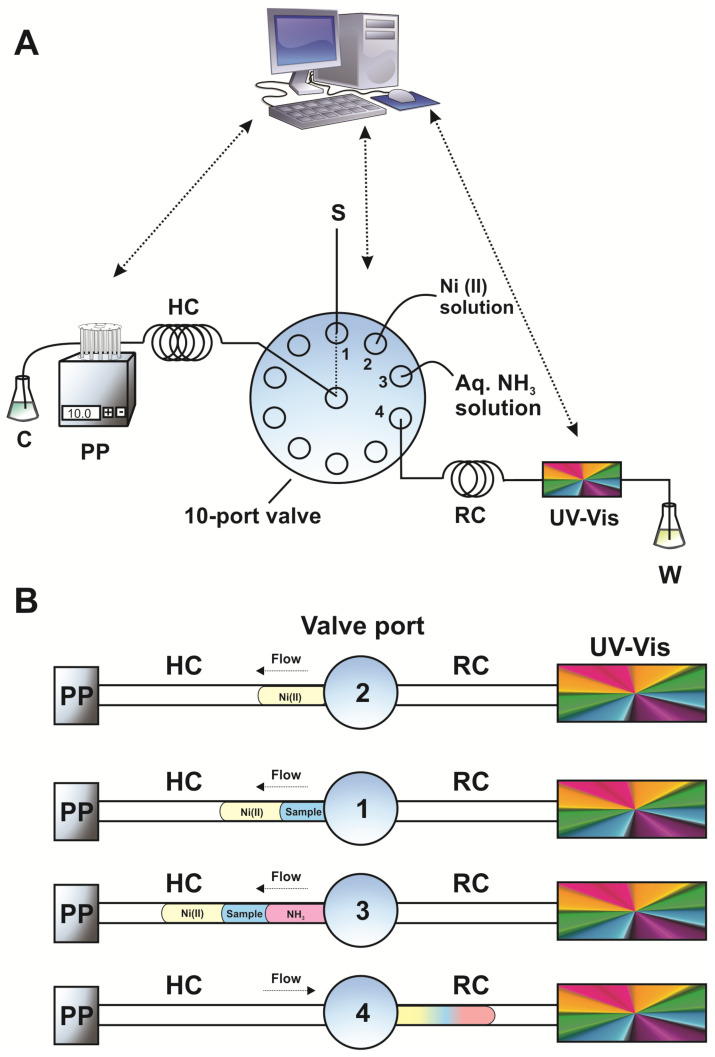
(**A**) Graphical depiction of the zone fluidics manifold and (**B**) the analytical sequence steps for the analysis of CAP; PP = peristaltic pump, HC = holding coil, S = sample, UV-Vis = spectrophotometric detector, RC = reaction coil, C = carrier (water), W = waste.

A schematic representation of the ZF sequence is depicted in Figure 5. In brief, volumes of 50 μL of Ni(II) (at concentration of 10 mmol L^−1^), 100 μL CAP-containing dissolution samples and 50 μL aqueous ammonia solution (at concentration of 0.15% *v/v*) were sequentially aspirated in the holding coil (HC). Filling, washing and aspiration steps were performed automatically through the homemade software (see Section 3.3). The reaction product was formed upon flow reversal and passage of the zones through a 30-cm long reaction coil (RC) at a flow rate of 0.9 mL min^−1^. Peak heights of the Ni(II)–CAP complex were recorded spectrophotometrically at 340 nm. More detailed description of the ZF steps are tabulated in Table 3. The sampling throughput was estimated to be 25 h^−1^.

### 3.5. USP HPLC Conditions

The mobile phase consisted of 550 mL MeOH and 450 mL H_2_O containing 0.5 mL of concentrated H_3_PO_4_ [21]. The flow rate was 1.0 mL min^−1^ and the detection wavelength 220 nm. The mobile phase was filtered under vacuum through 0.45 μm filters and degassed ultasonically.

### 3.6. Accuracy Profiles

The analytical method was validated by taking into consideration the total error (systematic and random errors) as per “*Société Française des sciences et techniques pharmaceutiques*” (SFSTP) harmonization guidelines [19]. This approach involves the construction of the commonly known accuracy profiles and the theory behind this methodology has been comprehensively described elsewhere [22]. The accuracy profile is a decision-making graphical tool that helps the analyst to decide about the validity of the analytical method. Specifically, the tolerance and the acceptability intervals are plotted together in the same graph. The accuracy profile combined several tolerance intervals at various concentration levels. The basic idea of this concept lays in the fact that the result X concentration obtained from an analytical procedure is different from the unknown “true value” μ of the analyzed sample and is expected to be less than an acceptable limit λ.

−λ < Χ − μ < λ or |X − μ| < λ

The acceptable limit λ depends on the objectives of the analytical method and is typically expressed as the target value. For example, λ can be 1–2% for bulk drug substances, 5% for the determination of API in finished products, 15% in bioanalysis, etc. In our case, the selection of the suitable response function (e.g., linear, with and without transformation, weighted, unweighted) was based on the accuracy profiles and the *β*-expectation tolerance interval (*β*-ΕΤΙ). The *β*-ETI parameter is the interval where it is anticipated that a proportion *β* of future measurements will be distributed among the acceptance limits ± λ [23,24,25]. The analytical method is considered to be valid when the *β*-ETI is included inside the range −λ and +λ. The expression of the analytical profile is given below:

$$\left[bias{\left(\%\right)}_{j}-Q_{t}\left(v;\frac{1+\beta }{2}\right)\sqrt{1+\frac{1}{pnB_{j}^{2}}s_{r,j}\;};bias{\left(\%\right)}_{j}+Q_{t}\left(v;\frac{1+\beta }{2}\right)\sqrt{1+\frac{1}{pnB_{j}^{2}}s_{r,j}\;}\right]$$

where

$$bias{\left(\%\right)}_{j}=\frac{{\hat{\mu }}_{j}-\mu_{Tj}}{\mu_{Tj}}\times 100,\;s_{rj}\frac{{\hat{\sigma }}_{W,j}^{2}\;+\;{\hat{\sigma }}_{B,j}^{2}}{{\hat{\mu }}_{j}}\times 100,\;B_{j}=\sqrt{\frac{\frac{{\hat{\sigma }}_{Β,j}^{2}}{{\hat{\sigma }}_{W,j}^{2}}+1}{n\frac{{\hat{\sigma }}_{Β,j}^{2}}{{\hat{\sigma }}_{W,j}^{2}}+1}}\;\nu =\frac{{\left(R+1\right)}^{2}}{R+\frac{1}{n\left(p-1\right)}+1-\frac{1}{pn^{2}}}$$

${\hat{\mu }}_{j}$ is the estimate of the mean results of the *j^th^* concentration level

${\hat{\mu }}_{T}$ is the unknown “true value”

*p* is the number of series

*n* is the number of the independent replicate per series

*Q_t_* (*ν*;$\;\frac{1+\beta }{2})$ is the *β* quantile of the *t*-Student distribution with *ν* degrees of freedom

${\hat{\sigma }}_{W,j}^{2}$ is the within series variance

${\hat{\sigma }}_{B,j}^{2}$ is the between series variance

## 4. Conclusions

A new automated zone fluidics-based method for the analysis of the dissolution profile of captopril formulations was developed and validated. The method is rapid, simple and does not require complicated procedures prior to detection, being advantageous compared to analogous non-separation assays. Detection of the CAP–Ni(II) complex at higher wavelengths (340 nm), compared to the USP-recommended UV method (205 nm), provided better selectivity and generic character against potential UV-absorbing excipients such as saccharin. The analytical figures of merit of the proposed analytical scheme enable its direct application to dissolution tests of captopril capsules with minimum sample preparation. The method was fully validated using the total error concept by constructing the accuracy profiles. The validation data confirmed its reliability for the intended quality control applications.

## Figures and Tables

**Table 1:** Validation results for the determination of CAP in the in vitro dissolution samples.

Validation Criteria			
Response function (linear regression)	Slope	Intercept (×10^3^)	*r* ^2^
(*k* ^a^ = 3; *m* = 7; *n* = 3) (5–120%)			
Day 1	3.5007	−1.3337	0.9986
Day 2	3.5055	1.257	0.9979
Day 3	3.4988	1.9391	0.9978
Precision (*k* = 3; *n* = 3)			
C (%)	*s*_r_ (%) ^b^	*s*_R_ (%) ^c^	
5	1.7	2.3	
10	1.1	1.6	
25	1.0	1.8	
50	1.3	1.5	
75	0.8	2.1	
100	0.74	1.4	
120	0.6	1.2	
Trueness (*k* = 3; *n* = 3)			
C (%)	Relative bias (%)		
5	−2.3		
10	+3.3		
25	+1.0		
50	+2.3		
75	+3.5		
100	+0.3		
120	+1.6		
Accuracy (*k* = 5; *n* = 3)			
C (%)	Relative *β*-ΕΤΙ (%)		
5	(−9.00, 4.33)		
10	(−1.77, 8.29)		
25	(−5.42, 7.34)		
50	(−1.81, 6.47)		
75	(−1.60, 5.41)		
100	(−5.59, 6.09)		
120	(−2.62, 5.74)		
Linearity (*k* = 3; *n* = 3; *m* = 7) (5–120%)			
Slope	1.026		
Intercept	1.026		
*r* ^2^	0.9999		
LOD (%)	1		
LLOQ/ULOQ (%)	5/120		

^a^*k*:*m* and *n* correspond to the number of experiments, calibration levels and replicates, respectively. ^b^ *s*_r_ (%): relative standard deviation under repeatability conditions. ^c^ *s*_R_ (%): relative standard deviation under intermediate precision.

**Table 2:** Comparison of the dissolution data of the proposed ZF method with USP HPLC [21].

Time (min)	% CAP Release (±SD) (Brand A)			% CAP Release (±SD) (Brand B)		
	**ZF**	**HPLC**	***p*-Value**	**ZF**	**HPLC**	***p*-Value**
**5**	30.4 (±2.9)	31.2 (±3.2)	0.769	27.3 (±3.0)	26.5 (±2.5)	0.803
**10**	60.8 (±2.7)	59.6 (±2.9)	0.636	63.7 (±5.8)	61.8 (±4.1)	0.675
**15**	85.9 (±5.1)	86.7 (±4.6)	0.853	88.9 (±5.7)	87.1 (±4.9)	0.706
**20**	95.7 (±2.3)	96.5 (±2.7)	0.722	92.6 (±4.9)	94.1 (±4.1)	0.712
**30**	99.3 (±3.1)	100.2 (±2.9)	0.738	100.5 (±3.5)	99.7 (±4.0)	0.811
**60**	101.7 (±3.4)	100.9 (±3.2)	0.786	98.6 (±2.3)	99.1 (±2.9)	0.830

**Table 3:** ZF sequence for the automated determination of captopril in the in vitro dissolution sample.

a/a	Time (*s*)	Valve Position	Pump Action	Flow Rate (mL min^−1^)	Volume (μL)	Action Description
1	1	2	Off	—	—	Selection of Ni(II) solution port
2	5	2	Aspirate	0.6	50	Aspiration of Ni(II) solution in the HC
3	1	1	Off	—	—	Selection of sample port
4	10	1	Aspirate	0.6	100	Aspiration of sample in the HC
5	1	3	Off	—	—	Selection of aqueous NH_3_ solution port
6	5	3	Aspirate	0.6	50	Aspiration of aqueous NH_3_ solution in the HC
7	1	4	Off	—	—	Selection of UV-Vis detector port
8	120	4	Deliver	0.9	1800	Deliver of the reaction mixture to the UV-Vis detector

## Data Availability

The data presented in this study are available on request from the corresponding authors.

## Sample Availability

Samples of the compounds are available from the authors.
